# Protein Intake and Disability Trajectories in Very Old Adults: The Newcastle 85+ Study

**DOI:** 10.1111/jgs.15592

**Published:** 2018-11-01

**Authors:** Nuno Mendonça, Antoneta Granic, Tom R. Hill, Mario Siervo, John C. Mathers, Andrew Kingston, Carol Jagger

**Affiliations:** ^1^ Institute for Ageing, Newcastle University Newcastle upon Tyne United Kingdom; ^2^ Human Nutrition Research Centre, Newcastle University Newcastle upon Tyne United Kingdom; ^3^ Institute of Health and Society, Newcastle University Newcastle upon Tyne United Kingdom; ^4^ AGE Research Group Institute of Neuroscience, Newcastle University Newcastle upon Tyne United Kingdom; ^5^ NIHR Newcastle Biomedical Research Centre in Ageing and Chronic Disease Newcastle University and Newcastle upon Tyne NHS Foundation Trust Newcastle upon Tyne United Kingdom; ^6^ Institute of Cellular Medicine, Newcastle University Newcastle upon Tyne United Kingdom

**Keywords:** protein, malnutrition, aged, 80 and over, very old, disability

## Abstract

**Objectives:**

To determine whether protein intake is associated with better disability trajectories in the oldest adults (≥85) and whether muscle mass and muscle strength would partially mediate this.

**Design:**

Prospective cohort study.

**Setting:**

Newcastle‐upon‐Tyne and North Tyneside, United Kingdom.

**Participants:**

Community‐dwelling older adults aged 85 at baseline (N=722).

**Methods:**

Protein intake was estimated using two 24‐hour multiple‐pass recalls at baseline. Disability was measured as difficulty performing 17 activities of daily living at baseline and 18, 36, and 60 months. Trajectories were derived using mortality‐adjusted group‐based trajectory modelling. The effect of protein intake (g/kg of adjusted body weight (aBW)/d) on disability trajectories was examined using multinomial logistic regression.

**Results:**

Participants had 4 distinct disability trajectories (between the ages of 85 and 90: constant very low (AT1), mild (AT2), moderate (AT3), and severe (AT4). Each unit increase in protein (g) per kg of aBW/d was associated with greater odds of AT1 (odds ratio (OR=7.97, 95% confidence interval (CI)=1.96–32.43, p = .004) and AT2 (OR=3.28, 95% CI=1.09–9.87, p = .03) than of AT4 over 5 years in models adjusted for selected covariates. Participants with protein intake of 1.0 g/kg aBW/d or more were more likely to belong to AT1 (OR=3.65, 95% CI=1.59–8.38, p = .009) and AT2 (OR=2.12, 95% CI=1.16–3.90, p = .01) than to AT4.

**Conclusion:**

Higher protein intake, especially 1.0 g/kg aBW/d or more, was associated with better disability trajectories in the oldest adults. These findings will inform new dietary strategies to support active, healthy ageing. **J Am Geriatr Soc 67:50–56, 2019.**

The oldest adults (≥85) are the fastest growing age group in most Western societies and are at high risk of disability. Disability is defined as difficulty maintaining the status quo in relation to the individual's basic functioning care and is measured according to activities of daily living (ADLs), such as the ability to feed oneself, bathe, dress, and transfer to and from the toilet. A more complex set of behaviors focused on the ability of an individual to preserve independence within the larger community (e.g., housekeeping and managing finances) is used in combination with ADLs and referred to as instrumental ADLs (IADLs). Difficulty performing ADLs and IADLs is associated with a number of adverse health outcomes, including mortality and poor quality of life.^1^, ^2^ The percentage of very old adults in England and Wales who require 24‐hour care is projected to increase by 82% from 2010 to 2030, resulting in need for an extra 63,000 care home places.^3^

Therefore, there is interest in slowing disability trajectories through modifiable risk factors, such as nutrition. Dietary protein is a sensible candidate because it may slow decreases in muscle mass and functional decline with aging.^4^, ^5^, ^6^ On average, protein intake is lower in older (66 ± 17 g/d) than younger (91 ± 22 g/d) adults^7^ because of multimorbidity, changes in oral health and taste perception, and loss of independence.^8^ For example, 28% of very old adults in northeast England had protein intake below the recommended dietary allowance (RDA) of 0.8 g per kg of adjusted body weight per day (g/kg aBW/d).^9^ Furthermore, the greater incidence and prevalence of multimorbidity in older adults can change protein requirements because of disease‐related tissue catabolism and inflammation.^10^, ^11^ The current protein RDA for all adults is based largely on short‐term nitrogen balance studies conducted in healthy young adults and does not take into account functional outcomes, such as disability.^12^ This has led others to propose that protein requirements are not the same for young and older adults.^8^, ^11^, ^13^ Whether adequate protein intake, mediated by better muscle strength, bone health, and physical function, can delay the onset of disability has been considered.^14^, ^15^

There is limited research on the association between protein intake and disability in older adults. Some observational studies have found that greater protein intake was associated with lower prevalence and incidence of disability in community‐dwelling participants,^5^, ^16^, ^17^ although these studies have limitations, including insufficient numbers of very old adults, use of a shorter disability scale (higher potential for floor and ceiling effects), failure to explore disabilities longitudinally, and failure to account for mortality. Our previous results have documented the prevalence of low protein intake in very old adults and the association with muscle strength,^6^, ^9^ but disability is a more relevant outcome for older people and for provision of care, and not all ADLs are necessarily mediated by muscle strength. To fill this gap, we aimed to determine the effect of protein intake on disability progression over 5 years in a large, sociodemographically representative cohort of 85‐year old individuals in northeast England. We hypothesized that protein intake would be associated with disability trajectory in the oldest adults and that muscle mass and muscle strength would partially mediate this.

## Methods

### Newcastle 85+ Study

Details of the Newcastle 85+ Study were previously published.^18^ Briefly, the Newcastle 85+ Study is a longitudinal population‐based study that approached all people turning 85 in 2006 (born in 1921) in Newcastle‐upon‐Tyne and North Tyneside, (UK) for participation. The recruited cohort was sociodemographically representative of the general U.K. population at the time and did not include individuals with end‐stage terminal illness.^18^ At baseline (2006–07), 722 community‐dwelling participants (60% women) had complete dietary intake data, body weight and height measurements, multidimensional health assessment (including disability), and complete general practice (GP) medical records. A flowchart of the recruitment and retention profile of the Newcastle 85+ Study is presented in Supplementary Appendix S1 and Supplementary Figure S1. This study was conducted according to the guidelines set out in the 1964 Declaration of Helsinki, and the Newcastle and North Tyneside local research ethics committee approved all procedures involving human subjects (06/Q0905/2). Written informed consent was obtained from all participants, and when that was not possible, consent was obtained from a caregiver or a relative according to the U.K. Mental Capacity Act 2005.

### Protein Intake Estimation

Complete details of the dietary intake assessment can be found elsewhere.^19^ Briefly, dietary intake was assessed according to 24‐hour multiple pass recall (24‐h MPR) on 2 nonconsecutive occasions at baseline. Energy and protein intake were estimated using McCance and Widdowson's sixth edition food composition tables.^20^ Body weight was adjusted to reflect a healthy (desirable) body mass index (BMI) in older adults of 22 to 27 kg/m^2^ and calculated as described previously^21^ (more details in 9). The protein RDA for all adults is 0.8 g/kg BW/d^22^, ^23^ but there are proposals to increase it to at least 1.0 g/kg BW/d for older adults.^8^ Accordingly, protein intake was expressed as g/kg aBW/d, below or above 0.8 g/kg aBW/d and below or above 1.0 g/kg aBW/d.^6^, ^9^

### Disability

A disability score was created by summing 17 self‐reported ADLs, IADLs, and mobility limitations, with each participant scoring 1 for each activity that could not be performed or was performed with any difficulty and 0 without difficulty. The disability score was calculated at baseline and after 18, 36, and at 60 months of follow‐up (Supplementary Figure S2). Ability to perform activities involving predominantly lower limb mobility (getting around the house, getting in and out of a chair, shopping for groceries, going up and down stairs, walking at least 400 yards (370 m)) was strongly related to Timed Up‐and‐Go test performance, which validated the self‐reported ADLs, IADLs and mobility items.^24^

### Baseline Socioeconomic, Lifestyle, and Health Factors

All baseline variables were assessed between June 2006 and October 2007. Participants were categorized into those who had spent up to 9 years, 10 to 11 years, or 12 or more years in full‐time education. We also categorized subjects into low (scores 0–1), medium (scores 2–6), and high (scores 7–18) physical activity groups based on a purpose‐designed and validated physical activity questionnaire^25^ and into those with none or at least one swallowing problem (including dry mouth). BMI was calculated as body weight (kg) divided by height^2^ (m). Fat‐free mass was estimated using a body fat analyzer (Tanita‐305, Tanita Corp., Tokyo, Japan). Disease count was created by scoring the most prevalent 7 chronic diseases as present (1) or absent (0) (cardiac, respiratory, and cerebrovascular disease; arthritis; hypertension; diabetes mellitus; and cancer in past 5 years)^26^; global cognition was assessed using the Standardized Mini‐Mental State Examination (SMMSE), with a score less than 26 indicating cognitive impairment; and depression was assessed using the 15‐item Geriatric Depression Scale (GDS). Serum albumin was measured using an automated version of the Bromocresol Green method.^27^

### Statistical Analysis

Group‐based trajectory models (GBTMs) were used to derive the optimum number of disability trajectories from age 85 to age 90. The model was extended to account for nonrandom participant attrition (predominantly mortality), with a link function for dropout probability according to age and based on previous response.^28^ Maximum likelihood was used to estimate the model parameters and mean disability count, followed a censored normal distribution. The optimum number of disabilities and model fit was assessed using the Bayesian Information Criteria and by confirming that the posterior probability of group membership was greater than 75%.^29^

For continuous variables, normality was tested using the Shapiro–Wilk test and confirmed using Q‐Q plots. Normally distributed data were presented as means and standard deviations, and non‐Gaussian distributed variables as medians and interquartile ranges. Categorical data were presented as percentages (with corresponding sample size).

The association between protein intake and disability trajectory was examined using multinomial logistic regression. Briefly, important variables were selected according to their clinical and theoretical relevance and univariate analysis with the disability trajectories. These variables were then fitted, removed, and refitted until the best possible but most parsimonious model was achieved while checking for model fit statistics throughout. Model 1 included only the intercept and protein intake (g/kg aBW/d) (continuous) or protein intake dichotomized at 1.0 g/kg aBW/d, Model 2 was further adjusted for sex and years spent in full‐time education, Model 3 included further terms for total energy intake and physical activity, and Model 4 was also adjusted for SMMSE score and number of chronic diseases. Missing values (physical activity, n = 1; years in full‐time education, n = 4; and SMMSE score, n = 1) were inputted with the mode (medium physical activity, 9 years spent in education) or the mean (SMMSE score 26.8) for the logistic regression models.

Disability trajectories were derived using Stata version 15.0 (package *traj*) (Stata Corp., College Station, TX) and the resulting estimates plotted using R version 3.2.2 (package *ggplot2*) (Statistics Department, University of Auckland, New Zealand). Most other statistical analysis were conducted using SPSS version 22.0 (IBM Corp., Armonk, NY). P < .05 was used to indicate statistical significance and point estimates (with confidence intervals (CIs)) to indicate clinical significance.

## Results

### Disability Trajectories

**Figure 1:**
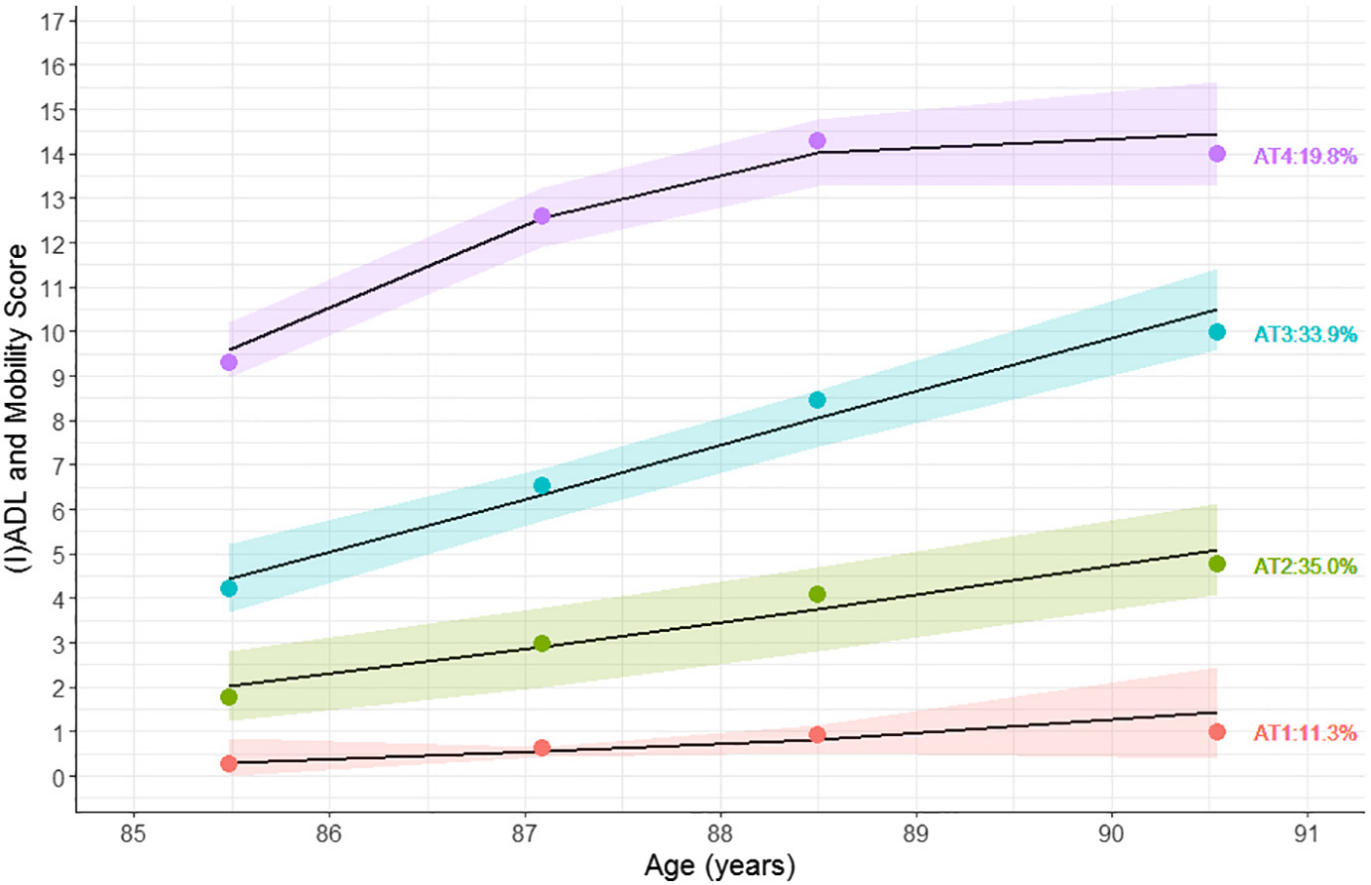
Disability trajectories with 95% confidence intervals of all participants. Percentages denote group sizes. Points are averages. Disability score was calculated by adding activity of daily living (ADL), instrumental activity of daily living (IADL), and mobility limitations. AT1=constant very low disability; AT2=low disability increasing to mild disability; AT3=mild disability increasing to moderate disability; AT4=moderate disability increasing to severe disability.

The disability trajectories (3 linear, 1 quadratic) were best represented using a 4‐group model. Trajectories are plotted in Figure 1 and described in detail in Supplementary Table S1. These trajectories were slightly different from previously derived disability trajectories in a different sample of the Newcastle 85+ Study.^26^ Briefly, participants had 4 distinct disability trajectories between the ages of 85 and 90: AT1, a constant very low disability trajectory (group size: 11.3%); AT2, low disability to increasing mild disability (group size: 35.0%); AT3, mild disability increasing to moderate disability (group size: 33.9%); and AT4, moderate disability increasing to severe disability (group size: 19.8%). All disability trajectories increased gradually with advancing age, except for the very low disability trajectory (AT1), whose participants had 0 or 1 disabilities at baseline and over 5 years. Those in the least favorable trajectory (AT4) had difficulty with a mean of 9 disability items at age 85 and progressed to 14 by age 90. The dropout rate (mostly through death) was also higher in trajectories with more disabilities. Three similar trajectories, each of which also revealed gradually increasing disability with advancing age, best represented disability trajectories for women (all linear trajectories) and men (2 linear, 1 quadratic trajectory) (Supplementary Figure S3). Because the trajectories were similar (but not the group sizes), we analyzed women and men combined to increase power to detect different trajectories.

### Socioeconomic, Lifestyle, and Health Factors Differed According to Disability Trajectories

More women, more participants with swallowing problems, those who had lost more than 5% of their body weight in the past 3 years, who did not drink alcohol, who were less physically active, who had greater cognitive impairment, who had more chronic diseases (e.g those in AT1 had, on average, 1 less chronic disease than those in the AT4), and who had higher GDS scores were in trajectories with greater disability (Table 1).

**Table 1:** Participant Characteristics According to Disability Trajectory

	Constant Very Low Disability, n = 74	Low Increasing to Mild Disability, n = 260	Mild Increasing to Moderate Disability, n = 244	Moderate Increasing to Severe Disability, n = 144
Female, n (%)	30 (40.5)	142 (54.6)	163 (66.8)	98 (68.1)
Body mass index, kg/m^2^, mean±SD	23.9±3.7	24.1±3.9	24.8±4.5	25.0±5.1
Fat‐free mass, kg, median (IQR)	48 (39–53)	44 (38–52)	42 (38–52)	42 (38–51)
Weight loss (≥5% in 3 years), n (%)	14 (27.5)	54 (32.7)	70 (48.6)	21 (56.8)
Years of full‐time education, n (%)				
0–9	47 (64.4)	156 (60.0)	157 (64.6)	98 (69.0)
10–11	15 (20.5)	70 (26.9)	53 (21.8)	32 (22.5)
12–20	11 (15.1)	34 (13.1)	33 (13.6)	12 (8.5)
Physical activity, n (%)				
Low	0 (0)	8 (3.1)	42 (17.2)	76 (52.8)
Medium	7 (9.5)	111 (42.9)	145 (59.4)	63 (43.8)
High	67 (90.5)	140 (54.1)	23.4 (57)	5 (3.5)
Alcohol drinker, n (%)	52 (85.2)	150 (78.5)	119 (72.6)	50 (56.2)
Total energy, MJ/d, median (IQR)	7.0 (6.0–8.8)	7.0 (5.9–8.6)	6.6 (5.6–7.9)	6.6 (5.3–8.3)
Total protein, g/d, median (IQR)	72 (53–89)	64 (51–78)	58 (46–72)	56 (48–73)
Total protein, g/kg of adjusted body weight per day, median (IQR)	1.04 (0.85–1.29)	1.00 (0.81–1.23)	0.91 (0.73–1.16)	0.94 (0.76–1.14)
<0.8	16 (21.6)	62 (23.8)	78 (32.0)	43 (29.9)
<1.0	32 (43.2)	125 (48.1)	147 (60.2)	86 (59.7)
<1.2	48 (64.9)	185 (71.2)	190 (77.9)	116 (80.6)
Swallowing problems, n (%)	34 (45.9)	140 (53.8)	144 (59.0)	98 (68.5)
Albumin, g/L, median (IQR)	41 (40–43)	41 (39–42)	40 (38–42)	40 (38–42)
Number of chronic diseases, mean±SD	1.6 (1.0)	2.1 (1.2)	2.4 (1.1)	2.6 (1.3)
Cognitively impaired, n (%)	7 (9.5)	41 (15.8)	51 (20.9)	66 (46.2)
Geriatric Depression Scale score, median (IQR)	1 (0–3)	3 (1–4)	3 (2–5)	5 (3–6)

Body weight was adjusted to nearest value to reflect healthy BMI in older adults aged 71 and older of 22–27 kg/m^2^, as described previously.^21^

Swallowing problems included dry mouth and difficulty swallowing for other reasons. Cognitive impairment was defined as having a standardized Mini‐Mental State Examination score less than 26.

SD=standard deviation; IQR = interquartile range.

### Association Between Protein Intake and Disability Trajectory

Participants with higher protein intake (g/kg aBW/d) at baseline were more likely to have a low (AT1) and mild (AT2) disability trajectory than a severe disability trajectory (AT4) in unadjusted models (odds ratio (OR)=3.23, 95% CI=1.41–7.36, p = .005), and in models adjusted for sex, education, total energy, physical activity, SMMSE score ,and number of chronic diseases (OR=.97, 95% CI=1.96–32.43, p = .004) (Table 2). This relationship was not apparent when protein intake was dichotomized at 0.8 g/kg aBW/d (current RDA for protein) (Supplementary Table S2), but it became evident for protein intake dichotomized at 1.0 g/kg aBW/d in unadjusted and fully adjusted models (Supplementary Table S3). Participants with protein intake of 1.0 g/kg aBW/d or greater were more likely to be in the low (AT1) (OR=3.65, 95% CI=1.59–8.38, p = .002) or mild (AT2) disability trajectory (OR=2.12, 95% CI=1.16–3.90, p = 0.015) than the severe disability trajectory (AT4). As a sensitivity analysis, models were also adjusted for free‐fat mass or grip strength. This adjustment decreased the association between protein intake and disability trajectories (e.g., participants with protein intake ≥1 g/kg aBW/d: AT1 vs AT4 (OR=3.04, 95% CI=1.26–7.35, p = .01) and AT2 vs AT4 (OR=1.88, 95% CI=1.00–3.54, p = .05)). Details of the sensitivity analyses with models further adjusted for interaction between physical activity and protein intake, adjusted for distribution of protein intake throughout the day or for quantity of protein per eating occasion, excluding missing cases, or stratified according to sex (Supplementary Table S4) are reported in Supplementary Appendix S1. No significant associations or change in coefficients of interest were detected.

**Table 2:** Association Between Protein Intake and Disability Trajectories in All Participants

	Model 1	Model 2	Model 3	Model 4
Trajectory	Odds Ratio (95% Confidence Interval) *P*‐Value			
Constant very low disability (n = 74)	3.23 (1.41–7.36) .005	2.47 (1.07–5.71) .03	6.96 (1.80–27.0) .005	7.97 (1.96–32.43) .004
Low increasing to mild disability (n = 260)	2.09 (1.09–4.00) .03	1.78 (0.93–3.43) .08	3.20 (1.10–9.34) .03	3.28 (1.09–9.87) .03
Mild increasing to moderate disability (n = 244)	0.93 (0.47–1.83) .83	0.89 (0.45–1.76) .73	1.44 (0.53–3.94) .47	1.49 (0.54–4.16) .44

Reference: moderate increasing to severe disability (n=144). Model 1 includes only the intercept and protein intake (grams per kg of adjusted body weight per day), Model 2 is further adjusted for sex and education, Model 3 includes further terms for total energy intake and physical activity, and Model 4 is also adjusted for Standardized Mini‐Mental State Examination score and number of chronic diseases.

## Discussion

### Main Findings

Consistent with our hypothesis, we showed that people aged 85.0 years old (± 0.5) with greater protein intake (g/kg aBW/d) were more likely to have fewer disabilities at baseline and shallower disability trajectories over the subsequent 5 years, after adjusting for covariates. These observations are unique because of the large number of very old adults, the wide array of disability measures, and the use of mortality‐adjusted GBTM. Theoretically, this would mean that a sustained increase in intake of 0.1 g of protein/kg aBW/d (7 g/d of protein for a 70‐kg individual, e.g., equivalent to 1 glass [200 mL] of semiskim milk) increased the chance of having a shallow disability trajectory (AT1) by 20% and a mild disability trajectory (AT2) by 13%. The protective effect was more pronounced in participants with protein intake of 1.0 g/kg aBW/d or more. This observation provides objective evidence in support of recommendations from expert groups such as PROT‐AGE and the European Society for Clinical Nutrition and Metabolism study group, who have proposed an increase in the protein RDA for older adults from 0.8 to 1.0 to 1.2 g/kg per day.^8^, ^11^

### Evidence from Other Studies

Our observations are consistent with those from a number of observational studies.^5^, ^16^, ^17^, ^30^, ^31^ For example, more than 110,000 postmenopausal women aged 50 to 70 from the Women's Health Initiative were followed for a mean of 11.5 years.^5^ Women with higher protein intake (measured at baseline using a food frequency questionnaire) (highest quintile: 1.19 ± 0.20 g/kg BW/d) had better self‐reported physical function and a slower rate of functional decline (assessed using the Medical Outcomes Study 36‐item Short‐Form Survey with 10 disability items).^5^ Another study found that older adults from the Health, Aging, and Body Composition Study (almost 2,000 community‐dwelling adults aged 70–79) with protein intake less than 1.0 g/kg aBW/d were at greater risk of developing mobility limitations over 6 years.^30^ Studies in different settings with different designs and shorter follow‐up periods have found more mixed results.^32^, ^33^ A 12‐week intervention trial with protein‐enriched foods and drinks in 75 older adults (mean age 76.8) followed for 6 months after hospital discharge did not show an effect of higher protein intake (112 g/d vs 78 g/d) on incidence of difficulty performing ADLs.^32^

Loss of muscle strength is related to selected functional limitations.^34^ A previous study found that participants in the Newcastle 85+ Study with higher protein intake (≥1 g/kg aBW/d) and high physical activity had the best performance on the grip strength test and less muscle strength decline over 5 years.^6^ The same effect was not present in those who had higher protein intake and low physical activity or low protein intake and high physical activity,^6^ suggesting that adequate protein combined with physical activity is required to optimally stimulate myofibrillar protein synthesis or at least reduce muscle strength loss in very old adults.^8^, ^11^ Our sensitivity analyses considered a possible interaction between protein intake and physical activity, but because of the small numbers of individuals with low physical activity and few disabilities (and high physical activity in those in the severe disability trajectories) this could not be inspected fully. We hypothesized that muscle mass and muscle strength partly mediated the observed association between protein intake and disability trajectories. In our sensitivity analyses, models were further adjusted for free‐fat mass or grip strength, and the coefficients changed only slightly, indicating that muscle mass and muscle strength only partially mediate the possible protective effect of high protein intake on disability trajectories in very old adults or that this is only on the pathway of certain disabilities, such as mobility limitations.

### Strengths and Weaknesses

The uniqueness of our approach lies with the large number of very old (sociodemographically representative) adults included in our study, the comprehensive multidimensional health data collected, and the large range of ADLs used (mobility items validated against Timed Up‐and‐Go test).^24^ Another major strength of the present investigation was the use of mortality‐adjusted GBTM to determine the relationship between protein intake at baseline and disability trajectories over 5 years. Attrition was high in this age group,^35^ and it is likely that failure to account for mortality resulted in biased trajectory group sizes.^28^

A limitation of our investigation is that some disability transitions might have not been captured because disability was assessed every 18 months (24 months in the last phase) over the 5 years of follow‐up. The model‐building strategy was comprehensive and adjusted for several important confounders, but because healthy behaviors (e.g., greater physical activity, healthier diet, not smoking) cluster together,^36^ it is possible that higher protein intake served as a proxy for healthy behavior(s) that were unaccounted for. Disabilities such as difficulty feeding, cooking a hot meal, or shopping for groceries can lead to lower food intake and, ultimately, to lower protein intake. Although protein intake was measured at baseline, and disabilities were measured prospectively over 5 years, these analyses are prone to reverse causality.

## Conclusions

In models adjusted for socioeconomic, health, and lifestyle factors, in the oldest adults, higher protein intake, especially 1.0 g/kg aBW/d or greater, was associated with a shallower disability trajectory over the following 5 years. The results support the consensus statements that protein intake in older adults should approximate 1.0 to 1.2 g/kg BW/d, which is within the acceptable macronutrient distribution range. As part of the PRevention Of Malnutrition In Senior Subjects in the European Union (PROMISS) project (http://www.promiss-vu.eu/ for more information), these results will inform development of dietary strategies to support healthy aging and be tested for effectiveness and cost‐effectiveness in a long‐term trial.

## Supporting information


**Supplementary Figure S1.** Flowchart of recruitment and cohort retention profile of the Newcastle 85+ Study according to the sample used.
**Supplementary Figure S2**. The 17 basic (BADL) and instrumental activities of daily living (IADL) and mobility items used to derive the disability score.
**Supplementary Figure S3.** Disability trajectories with 95% confidence intervals in women and men.
**Supplementary Table S1.** Description of disability and dropout trajectories in all participants.
**Supplementary Table S2.** Association between protein intake cut‐off of 0.8 g/kg aBW/d and disability trajectories in all participants (OR, 95%CI, p‐value).
**Supplementary Table S3.** Association between protein intake cut‐off of 1.0 g/kg aBW/d and disability trajectories in all participants (OR, 95%CI, p‐value).
**Supplementary Table S4.** Association between protein intake (g/kg aBW/d) and disability trajectories in women and men.
