# Supplementary material for: Protein Intake and Disability Trajectories in Very Old Adults: The Newcastle 85+ Study
Source: J Am Geriatr Soc. 2018 Nov 1;67(1):50–6. doi: 10.1111/jgs.15592 (PMC6334273; doi:10.1111/jgs.15592)
Supplement: Supplementary file 1 — Supplementary Figure S1. Flowchart of recruitment and cohort retention profile of the Newcastle 85+ Study according to the sample used. Supplementary Figure S2. The 17 basic (BADL) and instrumental activities of daily living (IADL) and mobility items used to derive the disability score. Supplementary Figure S3. Disability trajectories with 95% confidence intervals in women and men. Supplementary Table S1. Description of disability and dropout trajectories in all participants. Supplementary Table S2. Association between protein intake cut‐off of 0.8 g/kg aBW/d and disability trajectories in all participants (OR, 95%CI, p‐value). Supplementary Table S3. Association between protein intake cut‐off of 1.0 g/kg aBW/d and disability trajectories in all participants (OR, 95%CI, p‐value). Supplementary Table S4. Association between protein intake (g/kg aBW/d) and disability trajectories in women and men. [file JGS-67-50-s001.docx]

**Supplementary methods**

*Statistical analysis*

The disability trajectories for women and men were also derived by GBTM and assessed by the BIC. Multinomial logistic regression models were used to assess the associations between protein intake (g/kg aBW/d) and disability trajectories in women and men separately. Due to the lower number of observations in the stratified analyses, the models were as follows: Model 1 included terms for the intercept and protein intake (g/kg aBW/d) only (continuous) and Model 2 was further adjusted for energy intake and number of chronic diseases. Disability trajectories were derived using STATA v15.0 (package *traj*), resulting estimates were plotted with R v3.2.2 (package *ggplot2*), and multinomial logistic regression models were performed with the IBM statistical tool SPSS v22.0. P<0.05 was used as the cut-off for significance throughout and data are presented as point estimates and confidence intervals.

**Supplementary results**

*Sensitivity analyses – Models adjusted for physical activity interaction or for within day variation in protein intake or quantity of protein per meal or excluding missing cases or stratified by sex*

No participants with low physical activity at baseline had the low disability trajectory (AT1) which did not allow testing the interaction between physical activity categories and protein intake. However, the interaction term between protein intake and physical activity was not significant when the latter was included as a score instead of categories. Models were repeated with additional adjustment for protein intake variation between eating occasions (coefficient of variation), or for one meal with at least 20 g/ 25 g/ 30 g of protein but no significant associations or change in the coefficients were detected. Models were also repeated excluding the few missing cases and the results did not change. Depression (GDS) was not included in the model due to multicollinearity concerns with SMMSE as to perform the GDS, a SMMSE score ≥15 is required. We were also unable to adjust our models for weight-loss of more than 5% over 3 years because it would halve our sample size and decrease the power. Models fitted for women and men separately showed that higher protein intake (g/kg aBW/d) (continuous) was associated with increased odds of having a low to mild disability trajectory (WT1) in women (OR: 4.77, 95%CI: 1.35-16.85, p=0.015) but not in men despite these trajectories having fewer women (**Supplementary Figure S3** and **Supplementary Table S4**). However, the trajectories and the number of adjusted terms were different than in the all participants’ model due to the lower number of observations (**Supplementary Table S4**).

**Supplementary Figure S1.** Flowchart of recruitment and cohort retention profile of the Newcastle 85+ Study according to the sample used.


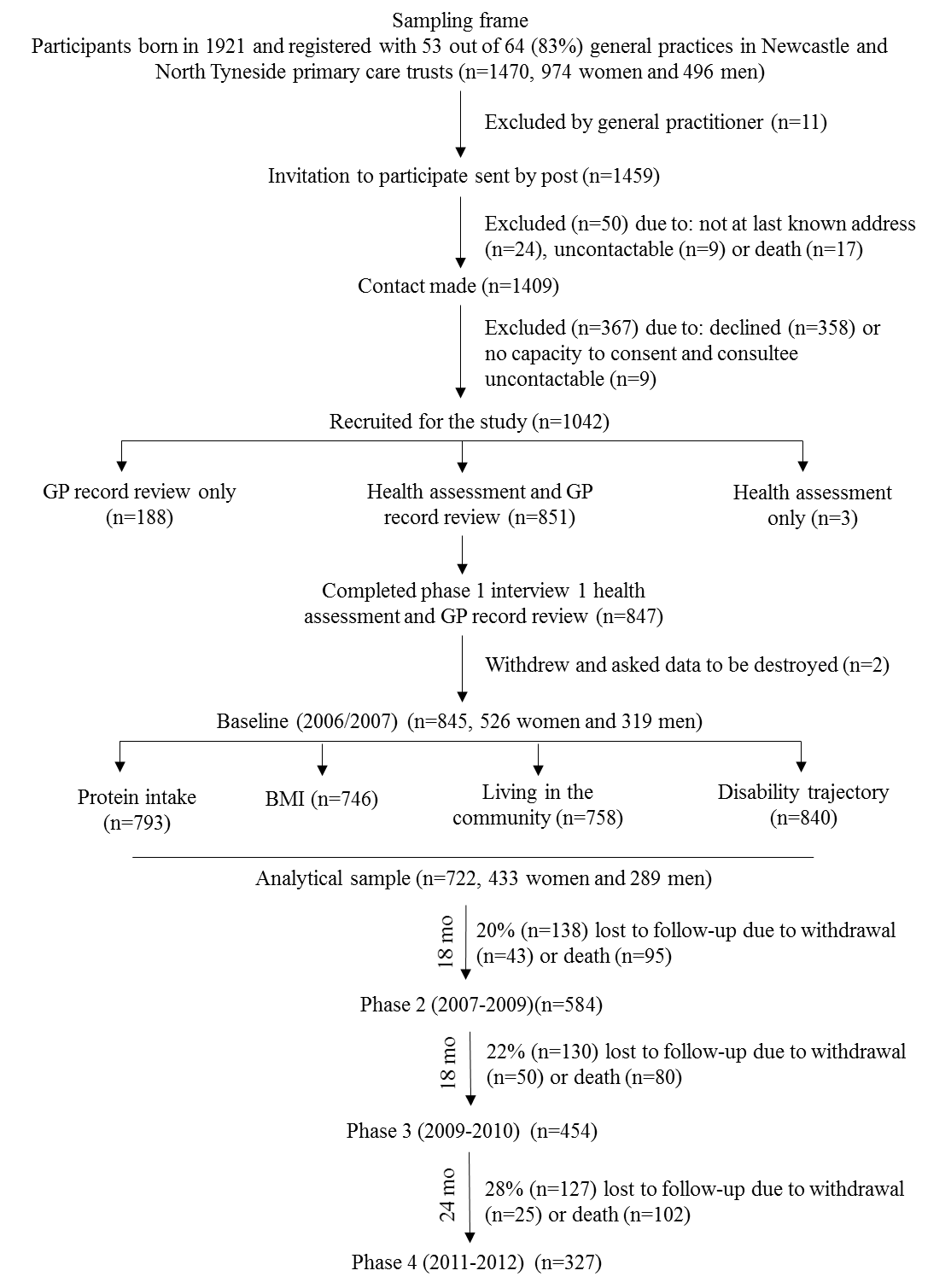


BMI, body mass index; GP, general practitioner; mo, months.

**Supplementary Figure S2**. The 17 basic (BADL) and instrumental activities of daily living (IADL) and mobility items used to derive the disability score.

| BADL | Are you able to get in and out of bed? |
| --- | --- |
|  | Are you able to get and out of a chair? |
|  | Are you able to get on and off the toilet? |
|  | Are you able to dress and undress yourself? |
|  | Are you able to wash your face and hands? |
|  | Are you able to wash yourself all over? |
|  | Are you able to cut your own toenails? |
|  | Are you able to feed yourself? |
| IADL | Are you able to cook a hot meal? |
|  | Are you able to shop for your groceries? |
|  | Are you able to do light housework? |
|  | Are you able to do heavy housework? |
|  | Are you able to manage money? |
|  | Are you able to manage your medications? |
| MOBILITY | Are you able to up and down stairs/steps? |
|  | Are you able to get around the house? |
|  | Are you able to walk 400 yards (370 metres) |

**Supplementary Figure S3.** Disability trajectories with 95% confidence intervals in women and men.


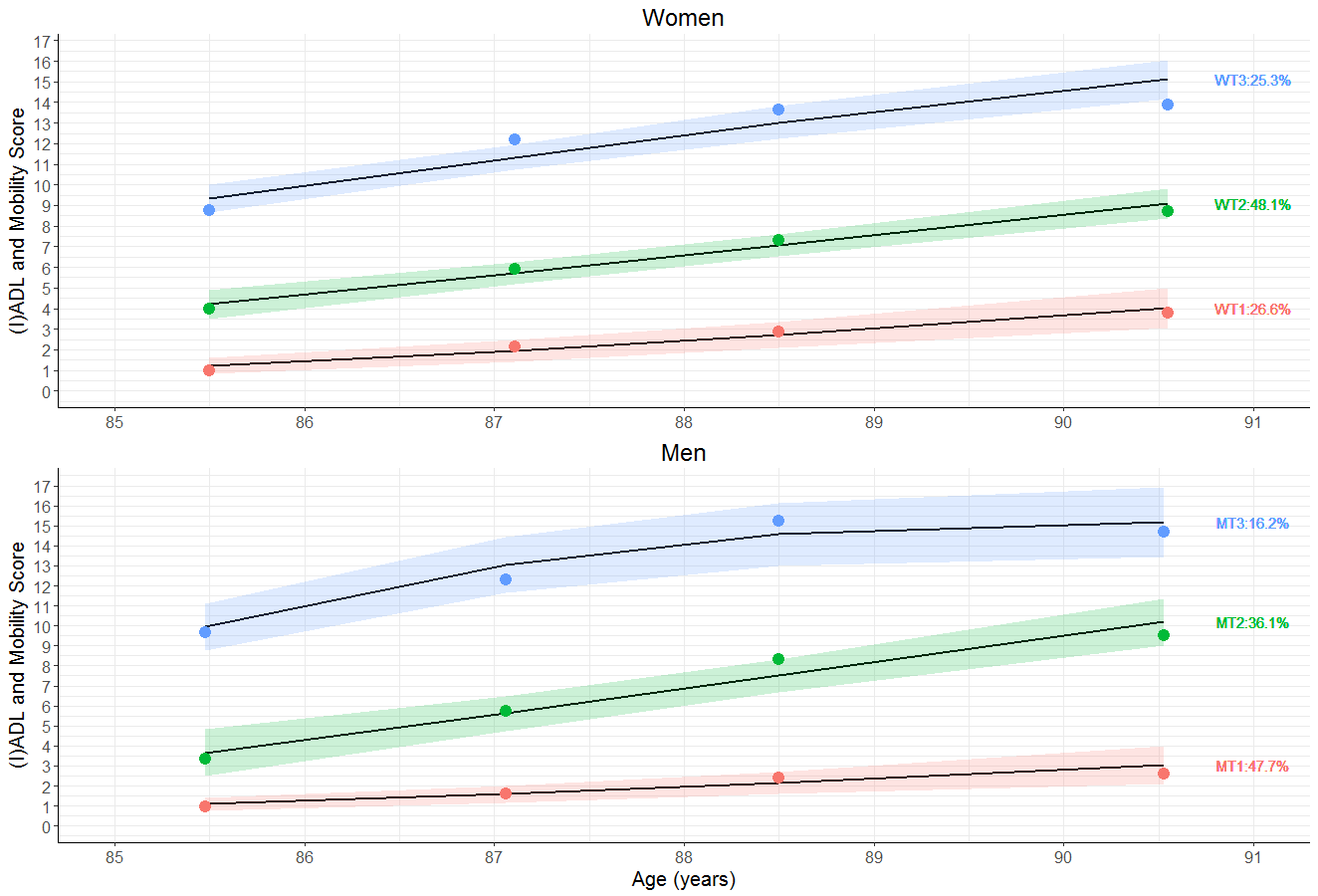


Percentages denote the group sizes. Points are averages. Disability score was calculated by adding ADLs, IADLs and mobility limitations. W/MT1, Low to mild disability; W/MT2, Mild to moderate disability; W/MT3, Moderate to severe disability. IADL, instrumental activities of daily living; MT, male trajectory; WT, female trajectory.

**Supplementary Table S1.** Description of disability and dropout trajectories in all participants.

| **Group name** | | **Group size (%)** | **Disability trajectory** | **Dropout trajectory** |
| --- | --- | --- | --- | --- |
| AT1 | Constant very low disability | 11.3 | 0-1 disabilities relatively constant from baseline to phase 4 | Dropout rate increased from 15% at phase 2 to 20% at phase 4 |
| AT2 | Low increasing to mild disability | 35.0 | Difficulty with 2 (I)ADLs, at age of 85 that steadily progressed to 5 by phase 4 | Similar dropout rate as AT1 |
| AT3 | Mild increasing to moderate disability | 33.9 | Mild disability at baseline (4 (I)ADLs) that progressed to 10 disabilities by phase 4 | Increase from 19% at phase 2 to 26% at phase 4 |
| AT4 | Moderate increasing to severe disability | 19.8 | Moderate difficulty with 9 items at baseline that rose to 14 at phase 4 | Dropout rate was greatest in this group and increased from 29% at phase 2 to 44% at phase 4 |

(I)ADLs, instrumental activities of daily living; AT, trajectory in all participants.

**Supplementary Table S2.** Association between protein intake cut-off of 0.8 g/kg aBW/d and disability trajectories in all participants (OR, 95%CI, p-value).

| **Trajectories** | **Model 1** | | | **Model 2** | | | **Model 3** | | | **Model 4** | | |
| --- | --- | --- | --- | --- | --- | --- | --- | --- | --- | --- | --- | --- |
| AT1 (n=74) | 1.54 | 0.80-2.98 | 0.197 | 1.38 | 0.71-2.69 | 0.347 | 2.03 | 0.82-4.99 | 0.125 | 2.04 | 0.81-5.21 | 0.135 |
| AT2 (n=260) | 1.33 | 0.84-2.10 | 0.219 | 1.24 | 0.78-1.97 | 0.356 | 1.59 | 0.84-3.00 | 0.158 | 1.54 | 0.79-2.97 | 0.204 |
| AT3 (n=244) | 0.91 | 0.58-1.42 | 0.665 | 0.88 | 0.56-1.38 | 0.579 | 1.13 | 0.63-2.02 | 0.677 | 1.09 | 0.60-1.99 | 0.772 |
| AT4 (n=144) | (ref.) | | | (ref.) | | | (ref.) | | | (ref.) | | |

<1.0 g/kg aBW/d is the reference category for protein intake. AT1, Constant very low disability; AT2, Low increasing to mild disability; AT3, Mild increasing to moderate disability; AT4, Moderate increasing to severe disability. Model 1 only includes the intercept and protein intake (g/kg a BW/d); Model 2 is further adjusted for sex and education; Model 3 includes further terms for total energy intake and physical activity; and Model 4 is also adjusted for SMMSE score and number of chronic diseases. CI, confidence interval; g/kg aBW/d, grams per kg of adjusted body weight per day; OR, odds ratio.

**Supplementary Table S3.** Association between protein intake cut-off of 1.0 g/kg aBW/d and disability trajectories in all participants (OR, 95%CI, p-value).

| **Trajectories** | **Model 1** | | | **Model 2** | | | **Model 3** | | | **Model 4** | | |
| --- | --- | --- | --- | --- | --- | --- | --- | --- | --- | --- | --- | --- |
| AT1 (n=74) | 1.95 | 1.10-3.43 | 0.022 | 1.67 | 0.93-2.98 | 0.084 | 3.09 | 1.39-6.88 | 0.006 | 3.65 | 1.59-8.38 | 0.002 |
| AT2 (n=260) | 1.48 | 0.98-2.24 | 0.061 | 1.34 | 0.88-2.04 | 0.169 | 1.86 | 1.04-3.33 | 0.037 | 2.12 | 1.16-3.90 | 0.015 |
| AT3 (n=244) | 0.98 | 0.64-1.49 | 0.919 | 0.93 | 0.61-1.43 | 0.746 | 1.20 | 0.70-2.05 | 0.517 | 1.36 | 0.78-2.39 | 0.277 |
| AT4 (n=144) | (ref.) | | | (ref.) | | | (ref.) | | | (ref.) | | |

<1.0 g/kg aBW/d is the reference category for protein intake. AT1, Constant very low disability; AT2, Low increasing to mild disability; AT3, Mild increasing to moderate disability; AT4, Moderate increasing to severe disability. Model 1 only includes the intercept and protein intake (g/kg a BW/d); Model 2 is further adjusted for sex and education; Model 3 includes further terms for total energy intake and physical activity; and Model 4 is also adjusted for SMMSE score and number of chronic diseases. CI, confidence interval; g/kg aBW/d, grams per kg of adjusted body weight per day; OR, odds ratio.

**Supplementary Table S4.** Association between protein intake (g/kg aBW/d) and disability trajectories in women and men.

| **Trajectories** | OR | 95% CI | p-value | OR | 95% CI | p-value |
| --- | --- | --- | --- | --- | --- | --- |
|  | **Model 1** | | | **Model 2** | | |
|  | **Women** | | | | | |
| WT1 (n=108) | 2.69 | 1.09-6.60 | 0.031 | 4.77 | 1.35-16.85 | 0.015 |
| WT2 (n=213) | 1.63 | 0.72-3.68 | 0.241 | 2.31 | 0.77-6.91 | 0.133 |
| WT3 (n=112) | (ref.) | | | (ref.) | | |
|  | **Men** | | | | | |
| MT1 (n=141) | 1.28 | 0.50-3.27 | 0.613 | 1.37 | 0.40-4.76 | 0.618 |
| MT2 (n=101) | 0.45 | 0.16-1.26 | 0.128 | 0.35 | 0.09-1.36 | 0.346 |
| MT3 (n=47) | (ref.) | | | (ref.) | | |

W/MT1, Low to mild disability; W/MT2, Mild to moderate disability; W/MT3, Moderate to severe disability. Model 1 only includes the intercept and protein intake (g/kg a BW/d); Model 2 is further adjusted for total energy intake and number of chronic diseases. CI, confidence interval; g/kg aBW/d, grams per kg of adjusted body weight per day; OR, odds ratio.
